# Integrative Physiology: Update to the Grand Challenge 2020

**DOI:** 10.3389/fphys.2020.00489

**Published:** 2020-05-15

**Authors:** Geoffrey A. Head

**Affiliations:** Baker Heart and Diabetes Institute, Melbourne, VIC, Australia

**Keywords:** physiology, omics, grand challenge, research translation, research opportunities

## Introduction

**Figure 1:**
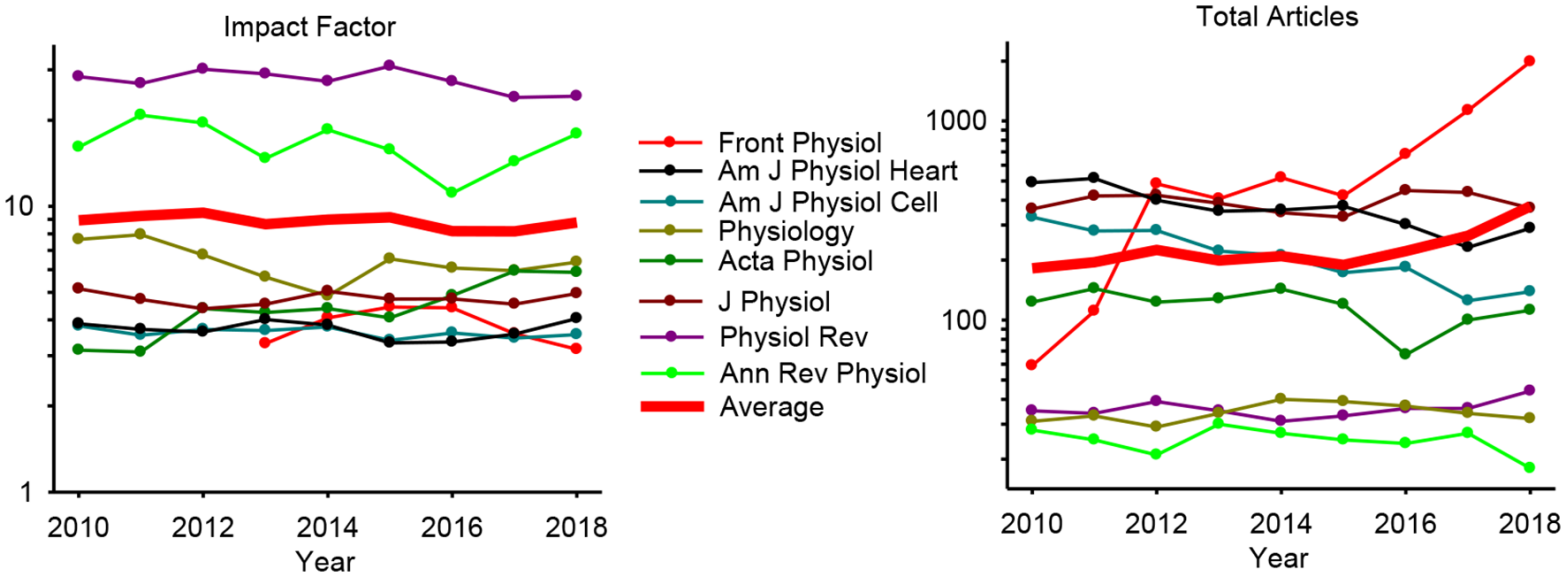
Year by year impact factor (**Left**) and total articles (**Right**) in 8 selected high-level physiology journals. The average trend line is shown in thick red.

After a decade has elapsed since I wrote the grand challenge for integrative physiology in 2010, it is fitting to not only reflect on how well we have progressed in this journey but also to consider the future challenges and opportunities that arise. It is also of interest to see how various physiology journals have been fairing in terms of impact factors and published articles. From a sample of eight prestigious physiology journals, it appears that the field of physiology itself has remained strong with no change or trend in average impact factor over this time (Figure 1). From a sample of 8 prominent physiology journals, the median impact factor of 4.5 in 2010 has increased to 5.4 some 9 years later and the average number of articles per journal has doubled from 182 to 373 (Figure 1). The standout improvers are Frontiers in Physiology that increased the number of published articles from 59 in 2010 to 1984 in 9 years and also Acta Physiologica that improved its impact factor nearly 2-fold from 3.1 in 2010 and to 5.9 in 2018. Interestingly, open access and general science journal PLOS ONE has declined in impact from 4.4 to 2.8 while increasing articles from 7000 to 18000. Journal of Neuroscience impact factor has also declined slightly from 7.2 to 6.0 as has the number of articles dropped from 1,700 to 800 in the same 9-year period. While these are anecdotal examples from other fields and limited evaluation in terms of quality and quantity, they do suggest that physiology at large has been doing quite well as a field over the last 10 years at least maintaining and, in some cases, improving.

### The Impact of “Omics” on Physiology

In my 2010 article discussing the grand challenge for integrative physiology, I emphasized new directions that had confronted physiologists related in particular to the genetic and “omics” revolution which had transformed biomedical research in the preceding years. The important question is whether we have adopted the challenge of the new information and technology or simply continued to generate research outcomes along our traditional lines. The large-scale investment in these platforms challenged traditional areas of endeavor such as physiology and has diverted the focus of biomedical research. However, there are many opportunities now and, on the horizon, to utilize the marvelous new technological developments which will hopefully spark physiologists' interest and create collaborative productivity. New models with tissue specific overexpression, constitutively active receptors and dominant negative genetics have been well-utilized and continue to expand our physiological understanding of complex systems and how they are affected by disease. There are also wonderful new technologies such as CRISPR/Cas9 for global, conditional and targeted gene editing and now *in-vivo* CRISPR/Cas9 gene editing. There have been 16 studies in Frontiers in Physiology since 2017 that have utilized this technology and interestingly none before that year.

High throughput genomics, proteomics, metabolomics, transcriptomics, nutrigenomics and more recently analysis of the human microbiome has given us a huge array of information that must also be contextualized within its physiological setting. Novel targets that are identified can be coupled with high throughput cellular screens to discover new therapies with physiological

regulatory potential. The discoveries of intracellular signaling, second messengers and regulatory influences such as post transcriptional modulation have taken our understanding of physiological processes to a new level. It is clear that physiologists that can integrate this information have a critical role in both the preclinical and clinical phases of discovery. However, the challenge will be to transform our thinking to embrace these new and quite marvelous opportunities. It is worth noting that in 2011 only 21 articles were published in Frontiers in Physiology that mention “omics” compared to over two thousand in 2019 which is a 10-fold growth. Interestingly, the omics field as determined by a PubMed search, has grown by 5-fold in that same period. Thus, we might conclude from this perhaps rudimentary analysis that physiologists at least in the Frontiers in Physiology journal, have embraced this new challenge with vigor. The major limitation however, is the amount of resources required to perform phenotypic analysis on all these new models and genetic variants. I suspect that we are not training and supporting sufficient physiologists to really cope with this wave.

### Microarray and GWAS Studies

A major advance in the hypertension field has been to use genetic risk scores to find genetic loci that contribute to high blood pressure. They combine the cardiovascular risk associated with variations in multiple genetic loci across the genome using genome-wide association study (GWAS). The major advantage of obtaining genetic risk scores using this method is that individual gene variants are less important and therefore the score is less influenced by imperfect linkages (Ehret, 2010). By and large however, such approaches have been disappointing as they explain a few percent of the overall cardiovascular risk (Head, 2016). The problems with such human studies are that the associations do not differentiate between genes that are changed due to high blood pressure and those that are causing it. Also, the genetic associations may well change as the course of the disease develops from the initiating phase to the structural and other changes that occur in vessels and the heart over many years (Ehret, 2010).

Experimental animal models of different diseases can be used to illuminate the mechanisms within tissue and systems that are not accessible in human studies. There are a number of rat and mouse strains for example that have been bred or genetically manipulated to develop high blood pressure. Strains such as the spontaneously hypertensive rat (SHR) and Schlager BPH mouse developed in the 1960's and 1970's, respectively have been widely used (Okamoto and Aoki, 1963; Schlager, 1974; Jackson et al., 2019). Studies from our laboratory suggested that the BPH mice had a neurogenic form of hypertension involving a much greater contribution of the sympathetic nervous system (SNS) (Davern et al., 2009). Marques and colleagues examined the hypothalamus of young and old BPH mice and compared them to the normotensive control BPN strain using gene array in 2011. While there were a number of genes associated with the development of hypertension, an unusual up and down pattern of expression of specific subunits of the GABA_A_ receptor was discovered (Marques et al., 2011a,b). Notably there was a lack of message for δ, α4 and β2 subunits particularly at 6 weeks of age when the hypertension was evident. To test the hypothesis that the overactive SNS was due to lack of GABA_A_ inhibitory signal in pre-sympathetic pathways, a GABA_A_ allosteric modulator benzodiazepine was administered chronically which had no effect on the blood pressure in the hypertensive mice but lowered blood pressure in the normal mice (Davern et al., 2014). This indicated that there was indeed a difference in the GABA_A_ receptors influencing blood pressure. By contrast, the neurosteroid allopregnanolone which is also an allosteric and expression modulator of GABA_A_ receptors had no effect in the normal mice but lowered blood pressure in the hypertensive mice (Stevenson et al., 2017). Importantly, the hypotensive action was associated with a restoration of the δ, α4 and β2 subunits expression in the hypothalamus and amygdala (Stevenson et al., 2017). Thus, a new potential therapeutic to treat hypertension has been revealed from the initial discovery using an exploratory microarray analysis (Head et al., 2019). Importantly, this therapy would target the SNS reactivity to stress which is not a mechanism that is targeted by current therapy modalities (Head et al., 2019).

### RNA-Sequencing

The development of RNA-sequencing has been a major step forward since it uses next generation sequencing to determine the transcriptome profile of any particular experimental or clinical scenario to reveal novel affected transcripts. The technique has the advantage over microarray in that it is limited to known genes. A recent review by Adeola et al. explored the implications of “omics” technology in the study of clock genes (circadiOmics) which encompasses the use of genomics, transcriptomics, proteomics and metabolomics (Adeola et al., 2019). In an excellent example using both RNA-seq and DNA arrays, Zhang and colleagues found that 43% of all genes were influenced by circadian rhythms (Zhang et al., 2014). The authors suggested that their study “highlights critical, systemic, and surprising roles of the mammalian circadian clock and provides a blueprint for advancement in chronotherapy.”

A recent advance has enabled RNA sequencing to be attributed to cells thus we can find populations of different cell types in a tissue with characteristic expression and in doing so, we can reveal rare cell populations and discover important regulatory relationships between genes. Thus, apparently histologically similar adjacent cells can have quite different expression profiles. Steven Potter has written an excellent review of single cell sequencing in development, physiology and disease (Potter, 2018). One example of note that piqued my interest in the capabilities of single cell RNA sequencing comes from Chen and colleagues who used this technique to reveal a much more complex cell diversity in the mouse hypothalamus than previously thought (Chen et al., 2017). They not only found the expected known neuropeptide and peptide combination containing neurons, they also found previously undescribed cell groups. Importantly, they went on to show that food deprivation affected the transcriptome of 7 of the 34 subtypes and in doing so uncovered cell types not previously associated with food intake (Chen et al., 2017). Thus, by using relatively simple physiological challenges one can reveal which cells respond and in what way they change their expression profile.

### MicroRNA

MicroRNAs (miRNA) are small non-coding RNAs that interact with the 3' untranslated region of specific RNAs to induce degradation (O'Brien et al., 2018). They can also induce translational repression. They are considered to be master regulators of gene expression and have been used as biomarkers since they are relatively stable and can be found in plasma (Roser et al., 2018). While their discovery was in 1993, they have increasingly been the focus of researchers interested in how gene expression is regulated during health and disease (Bhaskaran and Mohan, 2014). Importantly, discoveries in miRNA gene regulation offer the opportunity for novel therapy since mimics and inhibitors are now available and have been used *in vivo* (Bhaskaran and Mohan, 2014). There is one word of caution however, since the transfection may not exactly mimic the endogenous function (Jin et al., 2015). High concentrations may have non-specific consequences and even transfection at physiological concentrations may not induce changes in gene expression (Jin et al., 2015).

Marques and colleagues examined the differential expression of miRNA between kidneys of patients with high and normal blood pressure and found that miRNA-181a suppresses renin expression (Marques et al., 2011c). Renin expression was 6-fold higher in hypertensive kidneys and miRNA-181a levels 6-fold lower. *In vitro* studies showed that this miRNA bound to renin and regulated renin expression (Marques et al., 2011c). Interestingly, a similar renin-miRNA-181a pattern was discovered in the kidneys of the BPH hypertensive mouse where higher levels of renin were observed when levels of miRNA-181a were lowest (Jackson et al., 2013). This occurred at night when the mouse was most active and the SNS activity was highest (Jackson et al., 2013). During the day, there was no difference between the normotensive and hypertensive strains in either renin expression in the kidney or in miRNA-181a. One possibility for this difference between day and night might be that the miRNA is under the influence of the SNS and possibly circadian clock genes. Indeed, renal nerve denervation completely abolished the circadian differences in renin expression in the kidney, supporting this possibility.

We should not only consider 24-h patterns of expression but also longer periods such as might occur with aging. Yao and colleagues examined old and young human atrial tissue to identify how microRNA, genes and miRNA-mRNA interactions change with aging. They found 7 miRNA's, 42 genes and 114 pairs on miRNA-mRNA interactions differentially expressed (Yao et al., 2019). These types of studies are just the beginning to characterize how we age “genetically” and how these processes might be altered.

It is also of great interest that short-term interventions can alter miRNA levels. Yin and colleagues evaluated such a time-course in muscle-specific microRNA (miRNA) after rats ran uphill or downhill for 90 min (Yin et al., 2019). Interestingly, the miRNAs of interest were not affected by running uphill but were all increased after running downhill. Clearly some miRNAs are able to be regulated within the very short time frame of hours while others were induced after 48 h. These characteristics, once revealed for not only this type of intervention, but also other conditions such as stroke or myocardial infarction may be useful biomarkers and lead to a better understanding of mechanisms.

## Conclusion

In this review, I have touched on some of the opportunities that developments in omics and genetic technology have offered physiologists to explore. Clearly this is happening which is very pleasing to discover. I have highlighted only a few of the new techniques and examples that are now available. Really this is an extraordinary time in biomedical research. The first gene editing in humans using CRISPR/Cas9 for example is happening now (NCT03872479). The grand challenge as it was in 2010, will be for physiologists to be the translational link between the discoveries and the clinical trials. Importantly, we bring new insights and opportunities to our clinicians and pharmaceutical scientists. We must continue to build strong collaborations with our omics colleagues and utilize the new approaches to focus on mechanisms and regulatory functions that govern our physiological state and our health.

## Author Contributions

The author confirms being the sole contributor of this work and has approved it for publication.

## Conflict of Interest

The author declares that the research was conducted in the absence of any commercial or financial relationships that could be construed as a potential conflict of interest.
